# Cardiovascular Risk Factors after Childhood Cancer Treatment Are Independent of the FTO Gene Polymorphism?

**DOI:** 10.1155/2018/7495234

**Published:** 2018-02-20

**Authors:** Małgorzata Sawicka-Żukowska, Maryna Krawczuk-Rybak, Paweł Bernatowicz, Katarzyna Muszyńska-Rosłan, Jerzy Konstantynowicz, Włodzimierz Łuczyński

**Affiliations:** Department of Pediatric Oncology and Hematology, Medical University of Bialystok, Ul. Waszyngtona 17, 15-274 Bialystok, Poland

## Abstract

The study objective was to assess the prevalence of cardiovascular disease risk factors in patients treated for childhood cancer (*N* = 101) and to determine the involvement of clinical (cancer type and therapy) and/or genetic (*FTO* gene rs9939609 polymorphism) factors. Anthropometric features, laboratory findings, and standardized osteodensitometric indices (fat and lean mass) were considered. Overweight/obesity was found in 17.82% of the patients; however, central adiposity was found in as many as 42.5%. At least one abnormality in lipid metabolism was observed in 35.6%. Densitometry revealed elevated levels of fat mass in 44.55% of the patients. None of the parameters studied were associated with the *FTO* gene polymorphism. Standardized waist circumference was significantly higher in patients treated for leukemia than those treated for solid tumors (*p* = 0.04). Our findings indicate a high rate of central adiposity among childhood cancer survivors, especially leukemia patients. The prevalence of risk factors of cardiovascular disease after anticancer therapy is not *FTO* gene polymorphism-dependent.

## 1. Introduction

Childhood cancer survivors are at risk of developing overweight/obesity or cardiovascular disease [1], which can be due to central nervous system (CNS)/total body irradiation (TBI), administration of high steroid doses, reduced physical exercise (immobility), and changes in lifestyle/eating habits during and after treatment [2]. Obesity is a multifactorial phenomenon that, in the general population, is mainly caused by consumption of excessive amounts of energy and low physical activity. Thanks are due to novel diagnostic capabilities where risk factors of cardiovascular disease can be assessed using not only anthropometric parameters and lipid profiles but also by densitometry (including percentage of fat mass) [3]. The location of disease, disease advancement, and diverse therapeutic modes (including surgery, chemotherapy, and radiotherapy) are all factors that affect body mass in patients treated for childhood cancer. However, due to family histories of obesity, its genetic background is also investigated [4]. In recent years, the most commonly analyzed genetic factor is the fat-mass and obesity-associated *(FTO)* gene. The *FTO* gene rs9939609 polymorphism shows correlations with body mass index (BMI), overweight, obesity, and other risk factors of cardiovascular disease [5, 6]. While the mechanism of the *FTO* gene product has not been fully elucidated, it is assumed to play a role in the regulation of energy expenditure in the hypothalamus [7].

The aim of this study was to assess the prevalence of risk factors of cardiovascular disease in patients treated for childhood cancer and to determine the involvement of clinical (cancer type or therapy) and/or genetic (*FTO* gene polymorphism) factors. Anthropometric features (standardized: BMI and waist circumference), laboratory findings (lipid profile), and standardized osteodensitometric indices (fat and lean mass) were considered. The results were compared with the standards defined recently for Polish children [8–10].

## 2. Patients and Methods (Full Version in Supplemental Material)

A total of 101 childhood cancer survivors from the Department of Pediatric Hematology and Oncology of the Medical University of Bialystok (part of the Polish Pediatric Group for the Treatment of Leukemias/Lymphomas and Solid Tumors) were qualified to take part in the study during standard, periodic check-ups. The following parameters were evaluated in all subjects: anthropometric and disease-associated data, lipid profile, densitometric data, and genotype. The anthropometric parameters included age, sex, height, weight, BMI standardized deviation score (BMI-SDS), and waist circumference SDS. Underweight was defined as a BMI-SDS of ≤−1, normal weight as >−1 and <1, overweight as ≥1 and <2, and obesity as ≥2. Central obesity was recognized at a waist circumference above the 90th percentile [10]. The following disease-associated parameters were evaluated in the cancer survivor group: cancer type; disease duration; type of treatment regimen; elapsed time since therapy cessation; history of CNS radiotherapy or TBI (12 or 18 Gy); history of steroid treatment, type (prednisone and dexamethasone), and dose (standard or high); and history of hematopoietic stem cell transplantation (Table S1 in Supplementary Materials). Using densitometry, the following parameters were estimated and expressed in standard deviation scores: fat mass SDS (FAT SDS) and lean body mass SDS (LEAN SDS). Their values were compared with our own data and definitions developed by the International Society for Clinical Densitometry. Lipid profiles were analyzed in the hospital's central laboratory and included serum LDL cholesterol, HDL cholesterol, and triglyceride levels after 8 hours of fasting. Glucose and insulin concentrations were also assessed (fasting and 2 hours after oral glucose tolerance test). HOMA insulin resistance index was calculated according to the following formula: fasting insulin (microU/L) × fasting glucose (nmol/L)/22.5. All children were assessed for the *FTO* rs9939609 polymorphism via allelic discrimination with ABI 7900HT Fast Real-Time PCR System with SDS 2.1 software.

## 3. Presentation of Data and Statistical Analysis

Analysis results were presented as means with a standard deviation and rates of incidence of a given characteristic in the study group. *p* < 0.05 was considered to be statistically significant. Univariate analysis was performed using the Student's *t*-test in cases of continuous variables and the Chi-square test for nominal ones. Correlation of the obtained data was performed using the Spearman's test. Frequencies of the alleles observed in the control group were tested against the Hardy-Weinberg equilibrium using the Chi-square test.

## 4. Results

The study was conducted at the mean age of 12.7 years (±4.2), on average 8.2 (±3.5) years after termination of anticancer therapy. No differences were found between boys and girls in relation to age at examination and diagnosis or time elapsed since treatment cessation. However, patients treated due to lymphomas were significantly older than those treated for leukemias or solid tumors (*p* < 0.05) (see Table S2 in Supplementary Materials).

### 4.1. Results of Anthropometric Analysis

The mean standardized BMI was 0.69 ± 1.65. Overweight was found in 12 patients (11.88%), and obesity was found in 6 (5.94%). The mean standardized waist circumference was 1.07 ± 1.97. Central adiposity was recognized in 43 children (42.5%).

### 4.2. Laboratory Findings

At least one deviation in lipid metabolism was noted in 36 patients (35.6%). LDL cholesterol fraction and triglycerides were elevated in 29 (28.7%) and 9 (8.9%) patients, respectively. The HDL cholesterol fraction was decreased in 8 patients (7.9%).

Abnormal plasma glucose levels were noted in 2 patients (1.9%) after fasting and in 1 patient (0.9%) after oral glucose tolerance test. The insulin resistance index HOMA (mean 2.92 ± 10.4) was abnormal in 18 cases (17.8%) (HOMA > 3). Lipid and carbohydrate levels did not show any statistically significant differences with respect to sex and diagnosis. Children with insulin resistance had a higher standardized waist circumference compared to patients without insulin resistance (data not presented), but no difference was found in BMI-SDS. No differences were observed in other parameters between these groups of patients.

### 4.3. Results of Densitometry

Standardized fat mass (FAT SDS) was decreased (<−1) in 4 patients (3.96%) and increased (>1) in as many as 45 patients (44.55%). Standardized lean mass (LEAN SDS) was decreased (<−1) in 12 patients (11.88%) and increased (>1) in 21 patients (20.8%). No differences were noted in densitometric parameters according to sex and diagnosis (data not presented).

### 4.4. Results of Genetic Analysis

The distribution of *FTO* genotypes in the study group with regard to sex and diagnosis has been presented in Table 1. The distribution was consistent with the Hardy-Weinberg law. No statistically significant differences were found in the distribution of the respective *FTO* genotypes between the study group and our large reference group from the Podlaskie Province [6]. The numerical and percentage distribution of the respective *FTO* gene alleles in the study group has been presented in Supplementary Materials (Table S3).

The values of the standardized BMI according to genotype did not differ significantly (*p* = 0.77) and were as follows: AA, 0.94 ± 0.38; AT, 0.48 ± 0.24; and TT, 0.86 ± 0.31. The unfavorable *FTO* AA genotype was not significantly more common in the group of patients with overweight and/or obesity (Table S4 in Supplementary Materials). Likewise, no statistically significant differences were noted in the analysis of AA genotypes versus AT+TT or AA+AT versus TT (data not presented). The values of the standardized waist circumference did not differ according to *FTO* genotype (*p* > 0.05) and were as follows: AA, 1.07 ± 1.89; AT, 0.7 ± 1.94; and TT, 1.60 ± 1.88. Similarly, patients meeting the criteria of central adiposity did not show a higher frequency of the *FTO* AA genotype and A allele (data not presented).

Mean values of lipid profile and carbohydrate metabolism did not differ between groups of patients with the respective genotypes (Tables S5 and S6). Similarly, no differences were noted in the frequency of *FTO* genotypes in patients with abnormal lipids or carbohydrate metabolism compared to patients with normal parameters in this field (data not presented). The analysis of densitometric findings showed no difference in FAT and LEAN SDS according to *FTO* genotype (Table S7). No differences were found in the distribution of genotypes in patients with an elevated percentage of fat mass assessed by densitometry (FAT SDS) (data not presented).

### 4.5. Analysis of Risk Factors of Cardiovascular Disease with Respect to Clinical Features

#### 4.5.1. Primary Disease

Following the division into groups according to diagnosis, waist circumference SDS was significantly higher in the group of children treated for leukemia than in those treated for solid tumors (0.84 ± 0.07 versus 0.8 ± 0.02; *p* = 0.04).

#### 4.5.2. High Steroid Doses

The steroid dose applied to the body surface had no impact on the anthropometric, laboratory, and densitometric parameters studied (*p* > 0.05). The distribution of the respective genotypes and *FTO* gene alleles did not differ statistically in either group.

#### 4.5.3. CNS Irradiation

The anthropometric, laboratory, and densitometric parameters did not differ significantly between the group of children subjected to CNS irradiation and those who were not (*p* > 0.05). Considering the respective *FTO* genotypes, no statistically significant differences were found in anthropometric parameters, lipid profile, or densitometry between these groups.

#### 4.5.4. Stem-Cell and Marrow Transplantation

Patients who had undergone bone marrow or stem cell transplantation had significantly lower standardized BMI (−0.44 ± 0.91 versus 0.76 ± 1.67; *p* = 0.042) and waist circumference (−1.37 ± 0.80 versus 1.16 ± 1.95; *p* = 0.02) than those who had not. We did not observe a higher frequency of the unfavorable *FTO* genotype or its effect on laboratory parameters, body mass components, densitometry, or lipid profiles in the group of children after bone marrow transplantation.

#### 4.5.5. Diagnosis before, during, and after Puberty

In the group of children who developed cancer before puberty, no significant differences were found in the anthropometric parameters BMI-SDS and waist circumference SDS, apart from a significantly higher hip circumference expressed in SDS than patients who developed cancer during puberty or after its termination (1.05 ± 1.62 versus −0.45 ± 1.42; *p* > 0.05). Likewise, there were no differences in laboratory findings among these three groups of patients. In densitometry, LEAN SDS was significantly lower in children who developed cancer during or after puberty than in those who developed cancer before puberty (−1.50 ± 1.91 versus 0.51 ± 1.64, resp.; *p* = 0.01). No differences were noted in the distribution of *FTO* genotypes in these groups.

#### 4.5.6. Correlations

In the total study group, positive correlations were observed between standardized BMI (BMI-SDS) and standardized waist circumference (0.619; *p* = 0.0001). These correlations did not change in the analysis of subgroups with a different *FTO* genotype (data not presented). Analysis of densitometric data revealed strong correlations between some clinical parameters (e.g., standardized BMI) and densitometric findings (FAT) irrespective of the *FTO* genotype (Table 2).

Analysis of laboratory parameters revealed a positive correlation between BMI-SDS and level of triglycerides (0.258; *p* = 0.015), fasting insulin concentration (0.245; *p* = 0.02), and HOMA index (0.242; *p* = 0.02). A negative correlation was found between BMI-SDS and HDL (−0.328; *p* = 0.002). Similar correlations were obtained in the overweight and obesity groups.

No correlation was found between SDS-BMI or waist-SDS and clinical parameters, such as diagnosis, elapsed time since termination of treatment, and type of therapy (steroids, irradiation, and hematopoietic cell transplantation).

## 5. Discussion

Our findings indicate a high percentage of central adiposity among subjects treated for childhood cancer, especially after antileukemic therapy, although the percentage of obese/overweight survivors was not high. Neither standardized BMI nor waist circumference depended on other clinical features, including those associated with disease and treatment. No parameters were found to be affected by the *FTO* gene rs9939609 polymorphism. Thus, further research should be performed to determine the causes of central adiposity and attempts should be made for its prevention to reduce the risk of cardiovascular disease.

The reported rates of overweight and obesity among Polish children are 15.5% and 2%, respectively, similar to the rates found in our study [11]. Most authors observed higher percentages of overweight and obesity among patients after antineoplastic treatment [12]. However, it is not completely clear whether it is the disease, therapy, or patient lifestyle that is responsible for the increased risk of cardiovascular disease in these patients. The patient population most affected are those treated by total body irradiation [13], which, in extreme cases, may lead to considerable insulin resistance, type 2 diabetes, dyslipidemia, and hepatic steatosis [14]. Jarfelt et al. noted higher waist circumference in men treated for acute lymphoblastic leukemia with radiotherapy than male patients using other treatment methods. However, such differences were not observed in women [15]. Similar correlations were found in relation to body fat mass assessed using the DEXA method. The authors suggested the responsibility of growth factor deficiency due to CNS irradiation [15]. The study was conducted on a group of patients treated in the 1970s and 1980s using a dose of 24 Gy. Since that time, indications for CNS irradiation have been markedly limited [16], which resulted in a small number of irradiated patients in our study group (17 patients [16.83%] received 12 Gy). However, in the Janiszewski et al.'s study, obesity and central adiposity after leukemia treatment were not associated with CNS irradiation [17]. In our patient group, no differences were observed in the risk factors of cardiovascular disease according to treatment, including CNS and/or TBI. This data suggests that a 12 Gy dose does not have a major effect on the physical condition of patients, fatty tissue location, or lipid indices.

It is difficult to assess what factors determine the development of overweight and obesity in patients. In a study performed by Gofman and Ducore, a younger age at the time of diagnosis and Spanish origin correlated with the occurrence of obesity after anticancer treatment [18]; on the other hand, sex, irradiation and its dose, duration of therapy, and family history had no effect on body mass. Standardized BMI at the time of diagnosis of ALL also had a strong effect on standardized BMI after treatment and the risk of obesity [19]. Environmental factors play a key role in the development of obesity. In a small group of Brazilian patients, the prevalence rate of obesity was not higher after treatment for ALL and the role of environmental factors was suggested (i.e., living conditions of a developing country) [20].

As in other populations, the elevated BMI in patients after anticancer treatment is a predictor of insulin resistance [21]. However, insulin resistance after anti-ALL treatment was also observed in subjects with normal BMI [22]. In our study, we observed a high percentage of patients meeting the diagnostic criteria of insulin resistance (HOMA > 3). This observation and a high percentage of central adiposity indicate the risk of insulin resistance among patients after anticancer treatment.

Genetic factors also play an essential role in the pathogenesis of abnormal weight. Obesity in girls after leukemia treatment is associated with obesity in their mothers but not fathers [23]. It is still unknown which genes are strongly related to body mass. The *FTO* gene rs9939609 polymorphism is associated with body mass and risk of overweight and obesity in Polish children, as well as in the Podlaskie Province [6]. However, no relationship was found between the *FTO* gene polymorphism and body mass or risk of overweight/obesity in subjects after anticancer treatment. In Skoczen et al.'s study, a group of Polish children subjected to irradiation due to ALL showed a lower frequency of the T allele at the site of the *FTO* gene rs9939609 in subjects with overweight [24], which may be connected to a protective effect of the T allele preventing binge eating [25]. Polymorphisms of *FTO* and adiponectin genes can be used (in addition to age and BMI at time of diagnosis) in the model of obesity prediction after breast cancer treatment [26]. It is also known that obesity is associated with a higher risk of certain cancers, although no relation with the *FTO* gene polymorphism is known [27].

We found a high percentage of abnormal parameters of lipid metabolism, similar to other authors. In a group of 75 patients after ALL treatment, Gurney et al. showed abnormal levels of HDL cholesterol, but no other cholesterol fractions [28]. They observed a higher percentage of central adiposity in women than in the reference group, but not in the whole study group. Features of metabolic syndrome were found mainly in subjects undergoing CNS radiation. Likewise, a greater amount of fatty tissue, including total body fat, was revealed using the DEXA method in a group of individuals treated for ALL in childhood [15].

Lack of a control group is a limitation of the current study. However, we refer our findings to the database of healthy inhabitants of the Podlaskie Province. Future studies should include analysis of diet and physical activity of patients to determine the causes of central adiposity of subjects cured of leukemia. Similarly, other studies are characterized by limitations such as a small number of qualified patients, various inclusion criteria, or retrospective observation. Thus, it is still unknown whether the high frequency of risk factors of cardiovascular disease is actually caused by the disease and anticancer treatment [29].

## 6. Conclusion

The results of our study, including a high percentage of children with central adiposity, features of insulin resistance, abnormal lipid profile, and excessive amount of fatty tissue in densitometry (all without the genetic background of *FTO* gene polymorphism), indicate the potential threat of cardiovascular disease risk factors in childhood cancer patients. This patient population should be monitored by pediatricians and pediatric oncologists, especially to detect central adiposity in patients after antileukemic treatment. Proper diet, physical exercise, changes in lifestyle, and drugs in the case of failure should be recommended for the prevention and treatment of cardiovascular complications in this group of patients.

## Figures and Tables

**Table 1:** Distribution of the *FTO* gene genotypes in the study group by sex and diagnosis.

Study group	AA		AT		TT	
	*n*	%	*n*	%	*n*	%
Total	21	20.79	50	49.51	30	29.70
Girls	10	22.22	20	44.45	15	33.33
Boys	11	19.64	30	53.57	15	26.79
Diagnosis						
Leukemias	14	19.18	40	54.79	19	26.03
Lymphomas	3	21.43	4	28.57	7	50.00
Solid tumors	4	28.57	6	42.86	4	28.57

**Table 2:** Correlations between chosen densitometric parameters and standard BMI in the whole study group. ^∗^ *p* < 0.05 and ^∗∗^ *p* < 0.01.

	Height-SDS	BMI-SDS	FAT-SDS	LEAN-SDS
Height-SDS	—	0.220^∗^	0.345^∗∗^	0.666^∗∗^
BMI-SDS	0.220^∗^	—	0.789^∗∗^	0.414^∗∗^
FAT-SDS	0.345^∗∗^	0.789^∗∗^	—	0.330^∗∗^
LEAN-SDS	0.666^∗∗^	0.414^∗∗^	0.330^∗∗^	—

## Supplementary Materials

Supplementary Materials Supplemental Material includes full version of “Patients and Methods” with references, 7 tables, and with additional data for “Results” section. Table S1: type of treatment according to diagnosis. Table S2: study group characteristics with division into sex and type of cancer. Table S3: the frequency of the A and T alleles at the rs9939609 site of the FTO gene in the study group. Table S4: numerical and percentage distribution of FTO genotypes according to BMI. The differences were not statistically significant. Table S5: mean levels of lipid metabolism parameters with respect to the FTO genotype. Table S6: mean levels of carbohydrate metabolism parameters with respect to the FTO genotype. Table S7: osteodensitometric parameters with respect to the FTO genotype.

## References

[1] Oeffinger K. C., Mertens A. C., Sklar C. A. (2003). Obesity in adult survivors of childhood acute lymphoblastic leukemia: a report from the childhood cancer survivor study. *Journal of Clinical Oncology*.

[2] Green D. M., Cox C. L., Zhu L. (2012). Risk factors for obesity in adult survivors of childhood cancer: a report from the childhood cancer survivor study. *Journal of Clinical Oncology*.

[3] Brouwer C. A. J., Gietema J. A., Kamps W. A., de Vries E. G. E., Postma A. (2007). Changes in body composition after childhood cancer treatment: impact on future health status—a review. *Critical Reviews in Oncology/Hematology*.

[4] Pei Y. F., Zhang L., Liu Y. (2014). Meta-analysis of genome-wide association data identifies novel susceptibility loci for obesity. *Human Molecular Genetics*.

[5] Frayling T. M., Timpson N. J., Weedon M. N. (2007). A common variant in the *FTO* gene is associated with body mass index and predisposes to childhood and adult obesity. *Science*.

[6] Luczynski W., Zalewski G., Bossowski A. (2012). The association of the *FTO* rs9939609 polymorphism with obesity and metabolic risk factors for cardiovascular diseases in Polish children. *Journal of Physiology and Pharmacology*.

[7] Gerken T., Girard C. A., Tung Y.-C. L. (2007). The obesity-associated *FTO* gene encodes a 2-oxoglutarate-dependent nucleic acid demethylase. *Science*.

[8] Kulaga Z., Litwin M., Tkaczyk M. (2010). The height-, weight-, and BMI-for-age of Polish school-aged children and adolescents relative to international and local growth references. *BMC Public Health*.

[9] Konstantynowicz J., Nguyen T. V., Kaczmarski M., Jamiolkowski J., Piotrowska-Jastrzebska J., Seeman E. (2007). Fractures during growth: potential role of a milk-free diet. *Osteoporosis International*.

[10] Kulaga Z., Krzyzaniak A., Palczewska I., Barwicka K. (2007). Dynamika narastania nadwagi i otyłości dzieci i młodzieży - wybrana populacja polska na tle populacji USA. *Standardy Medyczne Pediatria*.

[11] Chrzanowska M., Łaska-Mierzejewska T., Suder A. (2013). Overweight and obesity in rural girls from Poland: changes between 1987 and 2001. *Journal of Biosocial Science*.

[12] Iughetti L., Bruzzi P., Predieri B., Paolucci P. (2012). Obesity in patients with acute lymphoblastic leukemia in childhood. *Italian Journal of Pediatrics*.

[13] Rajendran R., Abu E., Fadl A., Byrne C. D. (2013). Late effects of childhood cancer treatment: severe hypertriglyceridaemia, central obesity, non alcoholic fatty liver disease and diabetes as complications of childhood total body irradiation. *Diabetic Medicine*.

[14] Mayson S., Parker V., Schutta M., Semple R., Rickels M. (2013). Severe insulin resistance and hypertriglyceridemia after childhood total body irradiation. *Endocrine Practice*.

[15] Jarfelt M., Lannering B., Bosaeus I., Johannsson G., Bjarnason R. (2005). Body composition in young adult survivors of childhood acute lymphoblastic leukaemia. *European Journal of Endocrinology*.

[16] Stary J., Zimmermann M., Campbell M. (2014). Intensive chemotherapy for childhood acute lymphoblastic leukemia: results of the randomized intercontinental trial ALL IC-BFM 2002. *Journal of Clinical Oncology*.

[17] Janiszewski P. M., Oeffinger K. C., Church T. S. (2007). Abdominal obesity, liver fat, and muscle composition in survivors of childhood acute lymphoblastic leukemia. *The Journal of Clinical Endocrinology & Metabolism*.

[18] Gofman I., Ducore J. (2009). Risk factors for the development of obesity in children surviving ALL and NHL. *Journal of Pediatric Hematology/Oncology*.

[19] Zhang F. F., Rodday A. M., Kelly M. J. (2014). Predictors of being overweight or obese in survivors of pediatric acute lymphoblastic leukemia (ALL). *Pediatric Blood & Cancer*.

[20] Papadia C., Naves L. A., Costa S. S. S., Vaz J. A. R., Domingues L., Casulari L. A. (2007). Incidence of obesity does not appear to be increased after treatment of acute lymphoblastic leukemia in Brazilian children: role of leptin, insulin, and IGF-1. *Hormone Research in Pædiatrics*.

[21] Lowas S., Malempati S., Marks D. (2009). Body mass index predicts insulin resistance in survivors of pediatric acute lymphoblastic leukemia. *Pediatric Blood & Cancer*.

[22] Tonorezos E. S., Vega G. L., Sklar C. A. (2012). Adipokines, body fatness, and insulin resistance among survivors of childhood leukemia. *Pediatric Blood & Cancer*.

[23] Shaw M. P., Bath L. E., Duff J., Kelnar C. J., Wallace W. H. (2000). Obesity in leukemia survivors: the familial contribution. *Journal of Pediatric Hematology/Oncology*.

[24] Skoczen S., Bik-Multanowski M., Balwierz W. (2011). Homozygosity for the rs9939609T allele of the *FTO* gene may have protective effect on becoming overweight in survivors of childhood acute lymphoblastic leukaemia. *Journal of Genetics*.

[25] Wardle J., Llewellyn C., Sanderson S., Plomin R. (2009). The *FTO* gene and measured food intake in children. *International Journal of Obesity*.

[26] Reddy S. M., Sadim M., Li J. (2013). Clinical and genetic predictors of weight gain in patients diagnosed with breast cancer. *British Journal of Cancer*.

[27] Li G., Chen Q., Wang L., Ke D., Yuan Z. (2012). Association between *FTO* gene polymorphism and cancer risk: evidence from 16,277 cases and 31,153 controls. *Tumor Biology*.

[28] Gurney J. G., Ness K. K., Sibley S. D. (2006). Metabolic syndrome and growth hormone deficiency in adult survivors of childhood acute lymphoblastic leukemia. *Cancer*.

[29] Anarollo C., Airaghi L., Saporiti G., Onida F., Cortelezzi A., Deliliers G. L. (2012). Metabolic syndrome in patients with hematological diseases. *Expert Review of Hematology*.

