# Supplementary material for: Cardiovascular Risk Factors after Childhood Cancer Treatment Are Independent of the FTO Gene Polymorphism?
Source: Int J Endocrinol. 2018 Feb 20;2018:7495234. doi: 10.1155/2018/7495234 (PMC5838494; doi:10.1155/2018/7495234)
Supplement: Supplementary Materials — Supplemental Material includes full version of “Patients and Methods” with references, 7 tables, and with additional data for “Results” section. Table S1: type of treatment according to diagnosis. Table S2: study group characteristics with division into sex and type of cancer. Table S3: the frequency of the A and T alleles at the rs9939609 site of the FTO gene in the study group. Table S4: numerical and percentage distribution of FTO genotypes according to BMI. The differences were not statistically significant. Table S5: mean levels of lipid metabolism parameters with respect to the FTO genotype. Table S6: mean levels of carbohydrate metabolism parameters with respect to the FTO genotype. Table S7: osteodensitometric parameters with respect to the FTO genotype. [file 7495234.f1.docx]

PATIENTS AND METHODS - Supplemental Material

A total of 101 childhood cancer survivors from the Pediatric Hematology and Oncology Department of the Medical University of Bialystok (part of the Polish Pediatric Committee for the Treatment of Leukemia and Lymphoma and the Polish Pediatric Committee for the treatment of Solid Tumors) were qualified to take part in the study during standard, periodic check-ups. Antineoplastic treatment was performed in the department between 2001 and 2011. Study inclusion criteria were: Polish ethnicity, age 4–18 years, good general condition, diagnosis of cancer at 18 years of age or older, completed antineoplastic treatment with chemotherapy with or without steroids/radiotherapy, and lack of any symptoms of cancer relapse. Exclusion criteria were: age under 4 (because of inability to perform densitometry), central nervous system and/or bone tumors, presence of metal implants, and lack of consent for or cooperation during densitometry, psychiatric abnormalities (including nutritional disorders), evidence of chromosomal disorders during physical examination, hormonal or autoimmune disorders (celiac disease; disease of the thyroid, adrenals, and gonads) and hypostature. Diagnosis of specified cancer was based on cytochemical, morphological, and histopathological findings of bone marrow or peripheral blood or tissue, depending on the type of cancer. Subjects did not receive any medications. The study design was approved by the Ethics Committee of the Medical University of Bialystok in accordance with the Declaration of Helsinki (No. R-I-002/347/2009). The parents/guardians of each individual gave their consent for study participation.

*Anthropometric methods and additional assessments*

Anthropometric parameters (age, sex, height, weight, BMI standardized deviation score [SDS], waist circumference SDS) and lipid profile were evaluated in all subjects. Disease-associated parameters evaluated in the group of cancer survivors were: type of cancer; disease duration; treatment regimen used; elapsed time since therapy termination; use of radiotherapy of the central nervous system (CNS) or total body irradiation (TBI); history of steroid treatment, type (prednisone and dexamethasone), and dose (standard or high); and history of bone marrow transplantation (BMT) or peripheral blood stem cell transplantation (PBSCT). The protocols used in specific diseases are presented in Table S1.

The examination included measurements of height (cm, Harpenter’s stage-meter), weight (kg), waist circumference (cm), and blood pressure (mmHg) conducted by trained members of the study team. Height, weight, and waist circumference were measured in light sports garments or underwear, without shoes, and with an empty bladder. Each measurement was taken twice. Height and waist circumference were measured with a precision of 0.1 cm. With a difference in height measurements of >0.5 cm or in waist circumference of >3 cm, a third measurement was taken. Weight was measured with a precision of 0.1 kg; with results differing by > 0.3 kg, a third measurement was taken. BMI (kg/m^2^) and BMI-SDS values were calculated [1]. Considering BMI SDS, a BMI code was assigned for all study patients according to the formula: Code 1, normal BMI SDS from (-1) to (+1); Code 2, overweight – BMI-SDS = >1; Code 3, obesity – BMI-SDS = >2. Central adiposity was diagnosed based on waist circumference >90th percentile [2].

*Densitometric parameters*

During densitometry, the following parameters were estimated and expressed in the standard deviation score: standardized fat mass (FAT SDS) and standardized lean mass (LEAN SDS). The results were compared to the reference group from our center, assessed with the same device and method [3]. A standard deviation (FAT) >1 or ≤-1 was considered abnormal [4].

Lipid profiles were analyzed in the hospital’s central laboratory and included serum LDL-cholesterol, HDL-cholesterol, and triglyceride levels after 8 hours of fasting (local laboratory norms: LDL-cholesterol <2.6 mmol/L, HDL-cholesterol ≥1.1 mmol/L, triglycerides <1.7 mmol/L).

*Genotyping*

During blood sampling for the required diagnostic laboratory tests, a volume of 0.5 mL of blood was collected for genotyping. All children were assessed for the FTO rs9939609 polymorphism via allelic discrimination with ABI 7900HT Fast Real-Time PCR System with SDS 2.1 software (Applied Biosystems, Foster City, CA, USA). The A/T variant was evaluated with the use of validated commercially available probes included in the TaqMan SNP Genotyping Assay (Assay ID C__30090620_10, Applied Biosystems) with a dye (VIC/FAM, respectively) on the 5’ end. DNA was isolated from venous whole blood with the TaqMan Sample-to-SNP Kit (Applied Biosystems) according to the manufacturer’s instructions. The assay was conducted on 384-well plates in 5 μL; the mixture contained 1 μL of DNA, 2.5 μL TaqMan GTXpress Master Mix (Applied Biosystems), 0.25 μL TaqMan Genotyping Assay Mix 20x (Applied Biosystems), and 1.25 μL DNase-free water. The temperature profile of the reaction was as follows: initial denaturation at 95°C for 10 min; 40 consecutive cycles of denaturation at 92°C for 15 s; and hybridization/elongation at 60°C for 60 s. As contamination control, each reaction plate contained negative assays with water instead of DNA. A total of 99.3% of correct reactions were recorded.

Reference values for anthropometric measurements, densitometry, and laboratory tests were used based on the studies in the same center and of Polish origin [1, 3, 5].

*Data presentation*

Analysis results were presented as means with a standard deviation and rates of incidence of a given characteristic in the evaluated group of children. P<0.05 was considered to be statistically significant.

*Statistical analysis*

Univariate analysis was performed using the Student’s t-test in cases of continuous variables and the Chi-square test for nominal ones. Correlation of the obtained data was performed using the Spearman’s test. The frequencies of the observed alleles in the control group were tested against the Hardy-Weinberg equilibrium using the Chi-square test.

References

# 1. Z. [Kulaga](http://www.ncbi.nlm.nih.gov/pubmed?term=Kulaga%20Z%5BAuthor%5D&cauthor=true&cauthor_uid=20199693), M. [Litwin](http://www.ncbi.nlm.nih.gov/pubmed?term=Litwin%20M%5BAuthor%5D&cauthor=true&cauthor_uid=20199693), M. [Tkaczyk](http://www.ncbi.nlm.nih.gov/pubmed?term=Tkaczyk%20M%5BAuthor%5D&cauthor=true&cauthor_uid=20199693) et al., “The height-, weight-, and BMI-for-age of Polish school-aged children and adolescents relative to international and local growth references,” [*BMC Public Health*, vol. 10, article 109, 2010.](http://www.ncbi.nlm.nih.gov/pubmed/20199693)

2. Z. Kulaga Z, A. Krzyzaniak, I. Palczewska et al., “Dynamika narastania nadwagi i otyłości dzieci i młodzieży - wybrana populacja polska na tle populacji USA,” *Standardy Medyczne Pediatria*, vol. 4, no. 3, pp. 267-271, 2007.

3. J. Konstantynowicz, T. V. Nguyen, M. Kaczmarski et al., “Fractures during growth: potential role of a milk-free diet,” *Osteoporosis International*, vol. 18, no. 12, pp. 1601-1607, 2007.

4. The International Society for Clinical Densitometry Official Positions, 2007, <http://www.iscd.org/official-positions/official-positions/>, <http://www.iscd.org/wp-content/uploads/2012/10/ISCD2007OfficialPositions-Pediatric.pdf>

5. W. [Luczynski](http://www.ncbi.nlm.nih.gov/pubmed?term=Luczynski%20W%5BAuthor%5D&cauthor=true&cauthor_uid=22791637), G. [Zalewski](http://www.ncbi.nlm.nih.gov/pubmed?term=Zalewski%20G%5BAuthor%5D&cauthor=true&cauthor_uid=22791637) and A. [Bossowski](http://www.ncbi.nlm.nih.gov/pubmed?term=Bossowski%20A%5BAuthor%5D&cauthor=true&cauthor_uid=22791637), “The association of the FTO rs9939609 polymorphism with obesity and metabolic risk factors for cardiovascular diseases in Polish children,” *Journal of Physiology and Pharmacology*, vol. 63, no. 3, pp. 241-248, 2012.

| ALL | 72 | New York, ALL-IC-BFM 2002 |
| --- | --- | --- |
| AML | 1 | AML-BFM-2004-Interim |
| NHL T-cell | 5 | EURO-LB-02 |
| NHL B-cell | 5 | B-NHL BFM-04 |
| LGR | 4 | MVPP/B-DOPA |
| Neuroblastoma | 1 | Cojec |
| Nephroblastoma | 5 | SIOP |
| Germinal tumor | 3 | TGM-95 |
| Soft Tissue Sarcomas | 5 | CWS-2002, CWS-2005 |
| Soft Tissue Sarcomas | 5 | CWS-2002, CWS-2005 |

Table S1. Type of treatment according to diagnosis.

Table S2. Study group characteristics with division into sex and type of cancer.

|  | n | Mean age at analysis | Mean age at cancer diagnosis | Time that passed since treatment termination | | Mean age at anticancer treatment termination | |
| --- | --- | --- | --- | --- | --- | --- | --- |
| Total | 101 | 12.74 ±4.29 | 6.74 ±4.12 | 3.79 ±2.65 | | 8.25 ±3.53 | |
| Girls | 45 | 12.62 ±4.88 | 7.16 ±4.27 | 3.13 ±2.14 | | 8.96 ±4.00 | |
| Boys | 56 | 12.84 ±3.80 | 6.44 ±4.04 | 4.31 ±2.90 | | 7.77 ±3.11 | |
| Diagnosis |  | | | | | | |
| Leukemias (L) | 73 | 12.18 ±4.01 | 6.32 ±4.01 | 3.66 ±2.77 | | 8.19 ±3.42 | |
| Lymphomas(NHL) | 14 | 15.23 ±3.67 * | 9.45 ±4.12** | 4.13 ±2.14 | | 9.06 ±3.68 | |
| Solid tumors (ST) | 14 | 13.18 ±5.52 | 6.39 ±4.02 | 4.27 ±2.41 | | 7.42 ±5.07 | |
| Time of treatment termination |  | | | | | | |
| Up to 1 year | 17 | 13.02 ±4.48 |  | |  | |  |
| 1-5 years | 52 | 12.16 ±4.97 |  | |  | |  |
| Over 5 years | 32 | 13.54 ±2.67 |  | |  | |  |
| Control group | 633 | 14.06 ±3.27 |  | | | |  |
| Girls | 343 | 14.09 ±3.26 |  |  |  |  |  |
| Boys | 290 | 14.03 ±3.29 |  |  |  |  |  |

L – Leukemias

NHL – non-Hodgkin Lymphomas

ST - Solid Tumors

*p=0.009, p between L and NHL

**p=0.019, p between L and NHL

Table S3. The frequency of the A and T alleles at the rs9939609 site of the FTO gene in the study group.

|  | A (%)  N | T (%)  N |
| --- | --- | --- |
| Study group | 46 | 55 |
| Girls | 20 43.48% | 25 45.45% |
| Boys | 26 56.52% | 30 54.54% |
|  | p>0.05 | p>0.05 |
| Leukemias | 34 73.91% | 39 70.90% |
| Lymphomas | 5 10.87% | 9 16.36% |
| Solid tumors | 7 15.22% | 7 12.73% |
|  | p>0.05 | p>0.05 |

| Table S4. Numerical and percentage distribution of FTO genotypes according to BMI. The differences were not statistically significant. | | | | | | |
| --- | --- | --- | --- | --- | --- | --- |
|  | | | FTO rs9939609 | | | Total |
|  |  |  | AA | AT | TT |  |
| SDS BMI | normal | Numbers | 18 | 41 | 24 | 83 |
|  |  | % with BMI | 21.7% | 49.4% | 28.9% | 100.0% |
|  | overweight | Numbers | 1 | 7 | 4 | 12 |
|  |  | % with BMI | 8.33% | 58.33% | 33.33% | 100.0% |
|  | obesity | Numbers | 2 | 2 | 2 | 6 |
|  |  | % with BMI | 33.33% | 33.33% | 33.33% | 100% |
|  | overweight+obesity | Numbers | 3 | 9 | 6 | 18 |
|  |  | % with BMI | 16,66% | 50% | 33,33% | 100% |
| Total | | Numbers | 21 | 50 | 30 | 101 |

Table S5. Mean levels of lipid metabolism parameters with respect to the FTO genotype.

| FTO | | CHOL | HDL | LDL | Triglycerides |
| --- | --- | --- | --- | --- | --- |
|  | No | 21 | 20 | 18 | 20 |
| AA | Mean | 158.24 | 59.85 | 85.67 | 70.90 |
|  | SD | 26.853 | 13.589 | 24.384 | 27.728 |
|  | No | 47 | 47 | 44 | 45 |
| AT | Mean | 163.17 | 55.55 | 89.95 | 94.51 |
|  | SD | 41.376 | 13.070 | 38.474 | 58.337 |
|  | No | 29 | 28 | 27 | 28 |
| TT | Mean | 164.83 | 57.89 | 90.37 | 78.43 |
|  | SD | 26.184 | 16.102 | 23.825 | 47.282 |
|  | No | 76 | 75 | 71 | 73 |
| AT+TT | Mean | 163.80 | 56.43 | 90.11 | 88.34 |
|  | SD | 36.147 | 14.216 | 33.469 | 54.590 |
|  | p | 0.716 | 0.377 | 0.790 | 0.316 |

| Table S6.  Mean levels of carbohydrate metabolism parameters with respect to the FTO genotype.   \| FTO \| \| fasting glucose \| glucose after OGTT \| fasting insulin \| insulin after OGTT \| HOMA-IR \| \| --- \| --- \| --- \| --- \| --- \| --- \| --- \| \| AA \| no \| 21 \| 19 \| 21 \| 17 \| 21 \| \| Mean \| 87.19 \| 93.89 \| 8.73 \| 21.36 \| 1.97 \| \| SD \| 8.10 \| 10.32 \| 8.22 \| 16.19 \| 2.06 \| \| Median \| 88.00 \| 95.00 \| 6.10 \| 15.50 \| 1.43 \| \| AT \| no \| 47 \| 44 \| 46 \| 42 \| 45 \| \| Mean \| 88.32 \| 96.18 \| 8.56 \| 22.57 \| 1.93 \| \| SD \| 16.31 \| 14.44 \| 9.92 \| 17.05 \| 2.43 \| \| Median \| 89.00 \| 94.50 \| 4.53 \| 21.00 \| 1.01 \| \| TT \| no \| 29 \| 27 \| 27 \| 22 \| 27 \| \| Mean \| 85.97 \| 94.11 \| 8.13 \| 22.74 \| 1.71 \| \| SD \| 9.18 \| 10.87 \| 5.10 \| 16.52 \| 1.11 \| \| Median \| 88.00 \| 93.00 \| 8.00 \| 16.70 \| 1.56 \| \|  \| p \| 0.924 \| 0.943 \| 0.712 \| 0.907 \| 0.781 \|   Table S7.  Osteodensitometric parameters with respect to the FTO genotype.   \| Densitometric parameters \| FTO Genotype \| \| \| \|  \| \| --- \| --- \| --- \| --- \| --- \| --- \| \| AA \| AT \| TT \| AA+AT \| p \| \| LEAN SDS \| 0.14 ±1.31 \| 0.14 ±1.96 \| 0.53 ±1.88 \| 1.14 ±1.79 \| p>0.05 \| \| FAT SDS \| 1.69 ±2.04 \| 4.14 ±5.85 \| 5.35 ±7.16 \| 3.55 ±2.17 \| p>0.05 \| |  |  |  |  |  |
| --- | --- | --- | --- | --- | --- | --- | --- | --- | --- | --- | --- | --- | --- | --- | --- | --- | --- | --- | --- | --- | --- | --- | --- | --- | --- | --- | --- | --- | --- | --- | --- | --- | --- | --- | --- | --- | --- | --- | --- | --- | --- | --- | --- | --- | --- | --- | --- | --- | --- | --- | --- | --- | --- | --- | --- | --- | --- | --- | --- | --- | --- | --- | --- | --- | --- | --- | --- | --- | --- | --- | --- | --- | --- | --- | --- | --- | --- | --- | --- | --- | --- | --- | --- | --- | --- | --- | --- | --- | --- | --- | --- | --- | --- | --- | --- | --- | --- | --- | --- | --- | --- | --- | --- | --- | --- | --- | --- | --- | --- | --- | --- | --- | --- | --- | --- | --- | --- |
